# ADAM17 in tumor associated leukocytes regulates inflammatory mediators and promotes mammary tumor formation

**DOI:** 10.18632/genesandcancer.115

**Published:** 2016-07

**Authors:** Laura R. Bohrer, Thomas S. Chaffee, Pavlina Chuntova, Nicholas J. Brady, Patrice M. Witschen, Sarah E. Kemp, Andrew C. Nelson, Bruce Walcheck, Kathryn L. Schwertfeger

**Affiliations:** ^1^ Department of Lab Medicine and Pathology, University of Minnesota, MN, USA; ^2^ Microbiology, Immunology and Cancer Biology Graduate Program, University of Minnesota, MN, USA; ^3^ Department of Veterinary and Biomedical Sciences, University of Minnesota, MN, USA; ^4^ Masonic Cancer Center, University of Minnesota, MN, USA

**Keywords:** Breast cancer, inflammation, macrophage, ADAM17, Cox-2

## Abstract

The presence of inflammatory cells within the tumor microenvironment has been tightly linked to mammary tumor formation and progression. Specifically, interactions between tumor cells and infiltrating macrophages can contribute to the generation of a pro-tumorigenic microenvironment. Understanding the complex mechanisms that drive tumor cell-macrophage cross-talk will ultimately lead to the development of approaches to prevent or treat early stage breast cancers. As described here, we demonstrate that the cell surface protease a disintegrin and metalloproteinase 17 (ADAM17) is expressed by macrophages in mammary tumors and contributes to regulating the expression of pro-inflammatory mediators, including inflammatory cytokines and the inflammatory mediator cyclooxygenase-2 (Cox-2). Furthermore, we demonstrate that ADAM17 is expressed on leukocytes, including macrophages, within polyoma middle T (PyMT)-derived mammary tumors. Genetic deletion of ADAM17 in leukocytes resulted in decreased onset of mammary tumor growth, which was associated with reduced expression of the Cox-2 within the tumor. These findings demonstrate that ADAM17 regulates key inflammatory mediators in macrophages and that leukocyte-specific ADAM17 is an important promoter of mammary tumor initiation. Understanding the mechanisms associated with early stage tumorigenesis has implications for the development of preventive and/or treatment strategies for early stage breast cancers.

## INTRODUCTION

Breast tumor growth and progression require not only oncogenic changes within the epithelial cells, but also interactions between tumor cells and the stromal environment. Studies focusing on the tumor microenvironment have demonstrated that inflammation correlates with increased invasiveness and poor prognosis in breast cancer patients [1–3]. Inflammatory cells and their derived factors, including cytokines, chemokines and growth factors, which are important components involved in the normal wound healing response, are often present within the tumor microenvironment [4]. The presence of a wound healing environment in breast cancer has been linked to reduced overall survival [5], suggesting that this type of environment contributes to tumor progression.

Macrophages, which are key components of the inflammatory environment, have been implicated in breast cancer growth and progression [2, 6, 7]. Macrophages are recruited to the tumor microenvironment and reside within both the tumor parenchyma and the tumor reactive stroma and are associated with poor prognosis of breast cancer patients [1]. Macrophages produce protumorigenic factors in response to tumor-derived signals that in turn promote tumor cell proliferation, migration and invasion [8]. Macrophages also produce factors that alter the stroma, leading to enhanced angiogenesis and inhibition of anti-tumor immune responses [9–12]. In response to tumor cell derived factors, macrophages can produce both pro-inflammatory and anti-inflammatory factors, depending upon tumor stage and type, that act in combination to regulate tumor growth and progression [2, 13]. While numerous studies have focused on identifying key macrophage-derived factors that act on tumor cells to promote tumor growth and progression, less is known regarding the specific mechanisms that regulate the expression and release of these factors by macrophages.

The studies described here focus on a disintegrin and metalloproteinase 17 (ADAM17), also known as TNF-alpha-converting enzyme (TACE), which is a cell surface protease that has been implicated in the shedding of inflammatory cytokines and growth factors from the cell surface [14]. Studies have demonstrated that ADAM17 expression levels are increased in invasive breast cancers and correlate with poor prognosis [15, 16]. Mechanistically, ADAM17 on breast cancer cells has been found to cleave and release epidermal growth factor (EGF) family members from the cell surface, which then act through EGF receptor (EGFR) to promote tumor cell proliferation and invasion [16–18]. ADAM17 is also expressed on leukocytes and is known to contribute to inflammation through regulation of pro-inflammatory cytokines, such as TNFα, and adhesion proteins, such as L-selectin [19, 20]. ADAM17 is expressed by macrophages and studies have shown that ADAM17 regulates phagocytosis of apoptotic cells [21]. However, the contributions of ADAM17 to regulating macrophage function within the tumor microenvironment have not been examined.

We demonstrate here that ADAM17 is expressed by leukocytes, including macrophages, in mammary tumors and that genetic deletion of ADAM17 specifically in leukocytes leads to reduced formation of polyoma middle T (PyMT)-derived mammary tumors. Analysis of downstream effects of ADAM17 loss in macrophages demonstrates that ADAM17 regulates expression levels of the inflammatory mediator Cox-2, which has been previously shown to regulate tumor associated macrophage function [22–24]. Examination of ADAM17 and Cox-2 in human breast cancers using publically available databases demonstrates a link between increased expression levels of these factors and reduced relapse free survival, particularly in estrogen receptor (ER)-negative tumors. Together, these studies reveal novel functions for ADAM17 in the regulation of macrophage function in the microenvironment during tumor formation.

## RESULTS

### ADAM17 deletion in macrophages leads to reduced expression of inflammatory mediators

**Figure 1:**
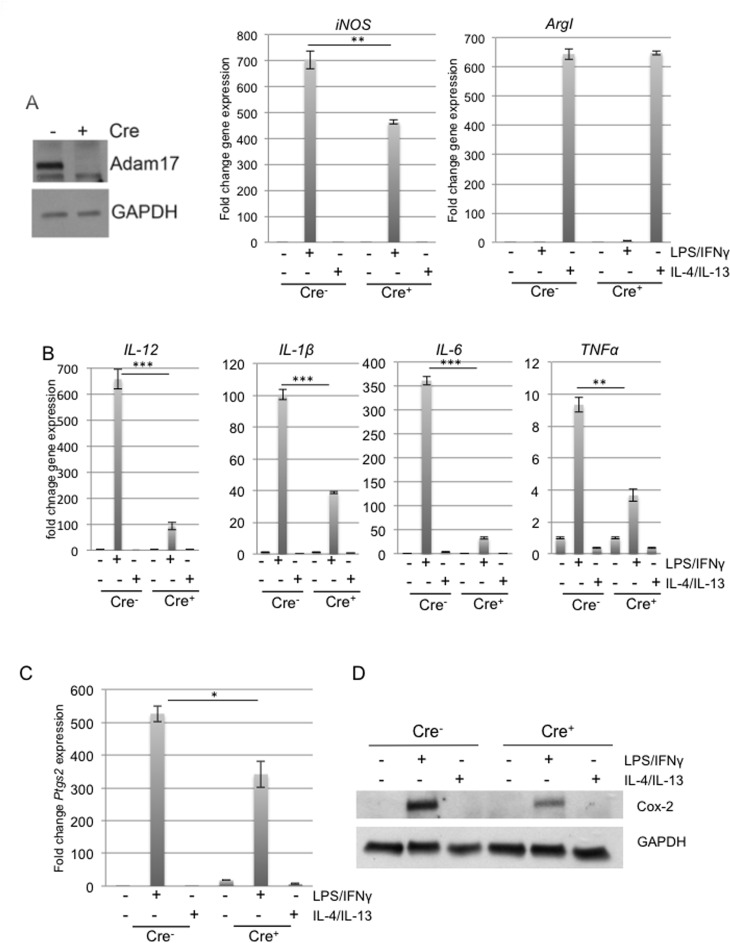
ADAM17 regulates expression levels of inflammatory mediators in macrophages A, B) Immunoblot analysis of BMDMs derived from ADAM17^WT^ and conditional-ADAM17^null^ mice was performed to detect ADAM17 expression. Analysis of GAPDH was performed to demonstrate equal loading. qRT-PCR analysis of BMDMs derived from ADAM17^WT^ and conditional-ADAM17^null^ mice following 18 hours of treatment with LPS/IFNγ or IL-4/IL-13. Expression levels of iNOS, ArgI, IL-12, IL-1β, IL-6 and TNFα were normalized to expression levels of CypB. C, D) BMDMs derived from ADAM17^WT^ and conditional-ADAM17^null^ mice were stimulated as described in panel B and Cox-2 expression was assessed by qRT-PCR analysis and immunoblot analysis. Gene expression levels were normalized to *CypB* and *GAPDH* was used to demonstrate equal protein loading. ^*^p<0.05, ^**^p<0.01, ^***^p<0.001.

Although ADAM17 has been linked to regulation of efferocytosis in macrophages [21], whether ADAM17 contributes to macrophage polarization in response to canonical polarizing stimuli has not been examined. To generate ADAM17-deficient macrophages, mice carrying a floxed ADAM17 allele (ADAM17^fl/fl^) were crossed with *Vav1-Cre* mice to generate conditional-ADAM17^null^ mice that lack ADAM17 expression in hematopoietic cells [25, 26]. The control mice used for these studies were ADAM17^fl/fl^ littermates that were negative for the Cre transgene (ADAM17^WT^). To determine whether ADAM17 contributes to macrophage polarization *in vitro*, macrophages were differentiated from the bone marrow of either ADAM17^WT^ or ADAM17^null^ mice. Immunoblot analysis revealed successful deletion of ADAM17 in Crepositive bone marrow derived macrophages (BMDMs) (Figure 1A). Initial studies were performed to determine whether ADAM17 regulates the expression of canonical inflammatory genes following exposure to either M1 (LPS/IFNγ) or M2 (IL-4/IL-13) stimuli. Expression of the canonical M1 target gene iNOS was reduced in the BMDMs lacking ADAM17 following M1 stimulation. However, expression levels of the M2 marker, ArgI, were not changed (Figure 1A). Based on these findings, we further assessed expression levels of canonical M1 markers, including the cytokines IL-12, IL-1β, IL-6 and TNFα, in BMDMs from ADAM17^WT^ and ADAM17^null^ mice. These cytokines were found to be reduced in macrophages lacking ADAM17 in response to LPS/ IFNγ stimulation (Figure 1B). To further examine the effects of ADAM17 deletion on inflammatory mediators, we assessed expression levels of Cox-2, which is a downstream target of pro-inflammatory mediators and a key regulator of tumor associated macrophage function [23, 24, 27]. Decreased expression of *Ptgs2*, the gene that encodes for Cox-2, and decreased Cox-2 protein expression were observed in the BMDMs lacking ADAM17 following exposure to LPS/IFNγ (Figure 1C,D). Together, these studies demonstrate that ADAM17 is a key regulator of pro-inflammatory mediators in macrophages in response to M1-polarizing stimuli.

### ADAM17 regulates expression of Cox-2 in macrophages following exposure to tumor cellderived factors

**Figure 2:**
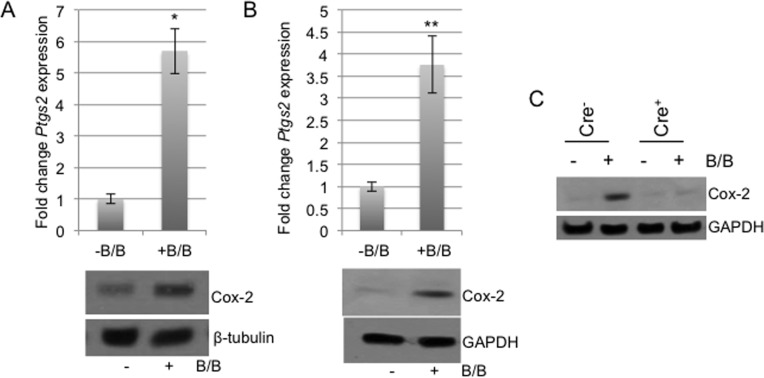
Tumor cell derived factors induce Cox-2 expression in macrophages in an ADAM17-dependent manner A, B) Conditioned medium was collected from HC-11/R1 cells following 24 hours of treatment with either B/B or ethanol as a solvent control. RAW 264.7 cells (A) or BMDMs (B) were exposed to the media for 18 hours prior to collection for qRT-PCR and immunoblot analysis to assess expression levels of Cox-2. Gene expression levels of *Ptgs2* were normalized to *CypB* and either β-tubulin or GAPDH was used to demonstrate equal protein loading. C) BMDMs derived from ADAM17^WT^ and conditional-ADAM17^null^ mice were stimulated as described in A and B and Cox-2 expression was examined by immunoblot analysis. GAPDH was used to demonstrate equal protein loading. ^*^p<0.05, ^**^p<0.01.

Cox-2 has been shown to regulate the protumorigenic functions of macrophages [23, 24, 27]. Based on our initial findings that ADAM17 regulates Cox-2 expression in response to inflammatory stimuli, further studies were performed to determine whether Cox-2 is induced in macrophages following exposure to tumor cell-derived factors. In previously published studies, we have utilized the HC-11/R1 mammary tumor cell line to examine the interactions between tumor cells and macrophages *in vitro* [28–30]. These cells express an inducible fibroblast growth factor receptor 1 (FGFR1) oncogene that is activated in response to treatment of cells with a synthetic dimerizer, B/B. To determine whether activation of FGFR1 in epithelial cells leads to production of soluble factors that alter Cox-2 expression in macrophages, HC-11/R1 cells were treated with either B/B or ethanol (solvent control). RAW 264.7 cells were exposed to conditioned media from these cells and Cox-2 expression was examined by qRT-PCR and immunoblot analysis. Both *Ptgs2* gene expression and Cox-2 protein expression were found to be increased in RAW 264.7 cells following exposure to iFGFR1-activated epithelial cells (Figure 2A). To confirm these findings, the studies were repeated using BMDMs, which verified that exposure to soluble factors derived from iFGFR1-activated cells led to increased expression of Cox-2 (Figure 2B). Based on the results from the LPS+IFNγ studies, we hypothesized that ADAM17 regulates Cox-2 expression in macrophages in response to tumor cell-derived factors as well. To examine this hypothesis, BMDMs from ADAM17^WT^ and ADAM17^null^ mice were exposed to tumor cell conditioned media and expression levels of Cox-2 in the BMDMs were examined by immunoblot analysis. As shown in Figure 2C, loss of ADAM17 in macrophages led to reduced Cox-2 expression in response to tumor cell conditioned media.

### ADAM17 expression on leukocytes and macrophages in PyMT mammary tumors

**Figure 3:**
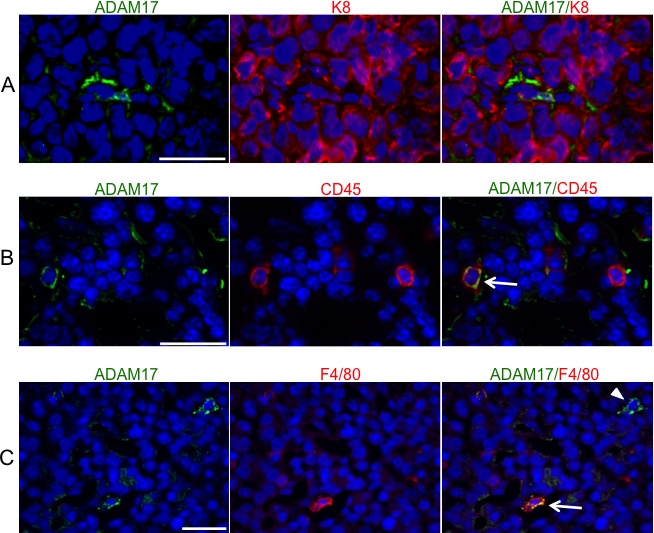
ADAM17 is expressed by leukocytes in mammary tumors A-C) Tumors were harvested from mice following orthotopic transplantation of MMTV-PyMT-derived tumor cells. Frozen tumor sections were stained with K8 and ADAM17 (A), CD45 and ADAM17 (B) and F4/80 and ADAM17 (C) to determine co-localization. Nuclei were stained with DAPI. Arrow indicates an F4/80^+^/ADAM17^+^ cell. Arrowhead indicates an F4/80^−^/ADAM17^+^ cell. Scale bars represent 25 μm.

Breast cancer cell-specific ADAM17 is known to contribute to tumor growth and progression [17, 18, 31]. ADAM17 is also expressed on leukocyte populations including macrophages and neutrophils and is known to contribute to inflammatory processes [21, 25, 32]. However, ADAM17 expression by leukocytes localized within mammary tumors has not been examined. To assess localization of ADAM17 expression, immunofluorescence analysis was performed on orthotopically transplanted mammary tumors. For these studies, primary mammary tumor cells derived from a mouse mammary tumor viruspolyoma middle T (MMTV-PyMT) transgenic mouse were orthotopically injected into mammary fat pads of FVB/N recipient mice. Resulting tumors were stained with antibodies to the luminal marker cytokeratin 8 (K8) and ADAM17 and imaged to assess ADAM17 expression and localization. Analysis of K8 staining suggested that the tumors are primarily luminal in origin, with K8 staining present throughout the tumor (Figure 3A). While low levels of ADAM17 staining were observed on tumor cells, higher levels of staining were observed on a number of K8-negative cells (Figure 3A). Further studies were performed to determine whether ADAM17 is expressed on tumor-associated leukocytes by co-staining tumors for ADAM17 and CD45. Analysis of co-staining revealed that approximately 47% of CD45^+^ leukocytes within the tumor express detectable levels of ADAM17 (Figure 3B, arrow). Because macrophages represent an important leukocyte population within PyMT tumors [33], further analysis was performed to determine whether F4/80^+^ macrophages express ADAM17. As shown in Figure 3C, approximately 45% of F4/80^+^ macrophages express detectable levels of ADAM17 (Figure 3C, arrow). We also observed a population of F4/80^−^/ADAM17^+^ cells (Figure 3C, arrowhead) suggesting that not all of the ADAM17^+^ cells are macrophages.

### ADAM17 deletion in leukocytes delays mammary tumor formation

**Figure 4:**
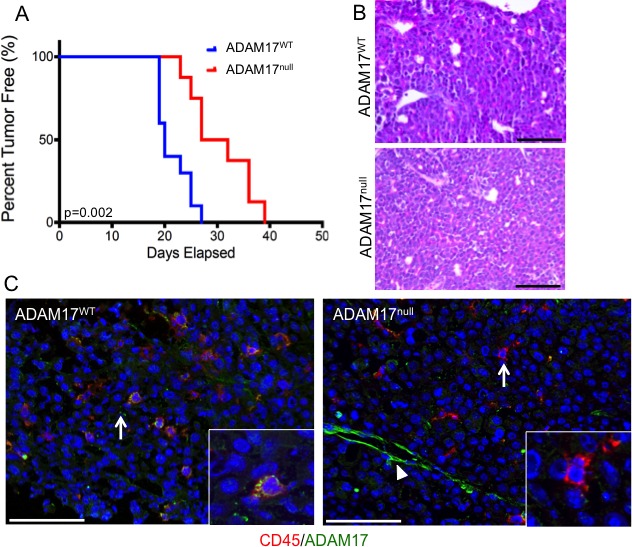
Loss of leukocyte-specific ADAM17 leads to decreased mammary tumor onset A) Primary tumor cells isolated from MMTV-PyMT mice were injected into the mammary fat pads of recipient ADAM17^WT^ and conditional-ADAM17^null^ mice and mice were monitored for tumor formation. n=10 (ADAM17^WT^) and 8 (conditional-ADAM17^null^). B) Initiating tumors from the indicated mouse genotypes [(n=6 (ADAM17^WT^) and 6 (conditional-ADAM17^null^)] were obtained at approximately 50 mm3 in size and examined for histology by H&E staining. C) Tumors were isolated from ADAM17^WT^ (n=3) and conditional-ADAM17^null^ (n=3) mice and frozen sections were stained for CD45 and ADAM17. Scale bars represent 50 μm.

Based on the observations that leukocytes in the tumor microenvironment express ADAM17, further studies were performed to determine whether leukocytespecific ADAM17 contributes to mammary tumor initiation and growth. PyMT cells were injected into the mammary fat pads of ADAM17^WT^ and conditional-ADAM17^null^ mice and evaluated for tumor onset and growth rate. Tumors developed significantly slower in the conditional-ADAM17^null^ mice compared with ADAM17^WT^ mice (Figure 4A). Once the tumors were established, tumor growth rates were not altered suggesting that once established, leukocyte-specific ADAM17 is dispensable for tumor growth (data not shown). Histological analysis of the tumors revealed that tumors from both genotypes consisted of expansive sheets of malignant cells without significant acinar formation (Figure 4B). The cytomorphology was similar and consistent with prior descriptions of the MMTV-PyMT model [34] and did not differ significantly between genotypes. Further immunofluorescence analysis demonstrated loss of ADAM17 in CD45^+^ cells of conditional-ADAM17^null^ mice compared with ADAM17^WT^ mice (Figure 4C, arrows and insets). Non-CD45^+^ cells retained ADAM17 expression (Figure 4C, arrowhead), further confirming staining and gene deletion specificity.

**Figure 5:**
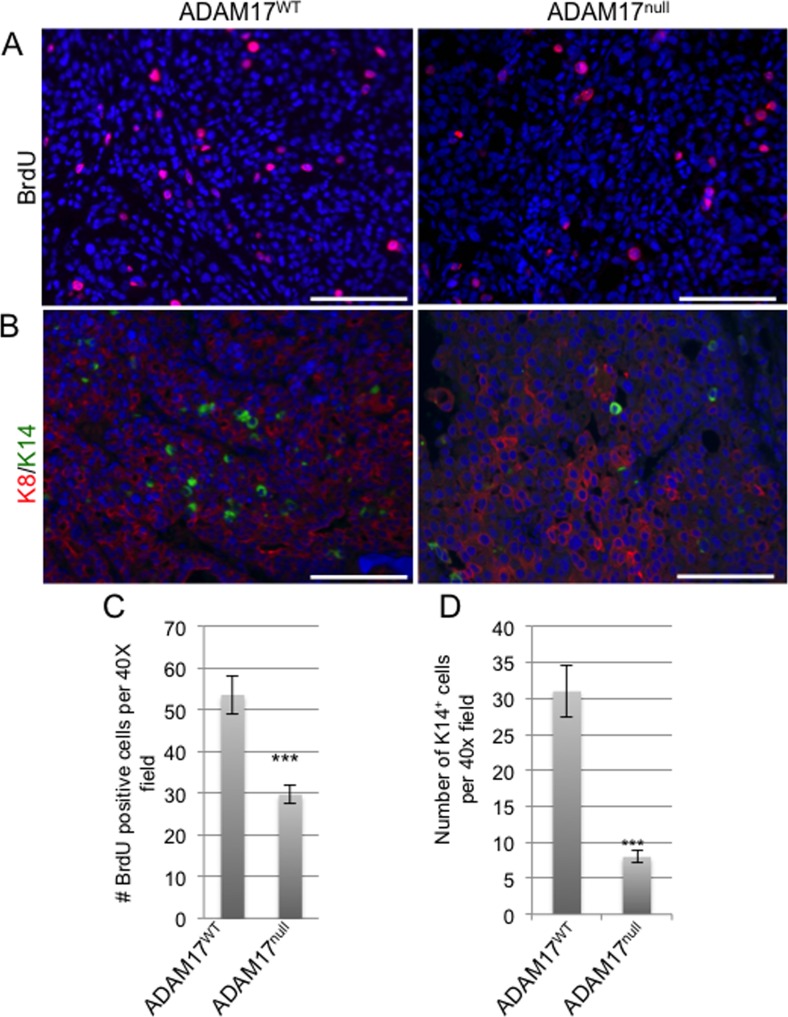
ADAM17 deletion in the hematopoietic compartment leads to reduced proliferation in developing tumors A) Initiating tumors from the indicated mouse genotypes were obtained at approximately 50 mm^3^ in size and proliferation was examined by assessing BrdU incorporation (n=3 tumors per genotype). B) Tumors were stained with the luminal epithelial marker K8 and the basal marker K14 (n=3 tumors per genotype). C) Quantification of BrdU-positive cells. D) Quantification of K14 positive cells. Scale bars represent 50 μm. ^***^p<0.001.

To determine whether decreased tumor onset correlated with altered tumor cell proliferation, early stage tumors were isolated at approximately 50 mm^3^ for analysis. Examination of proliferation demonstrated reduced BrdU incorporation in tumors growing in the conditional-ADAM17^null^ mice (Figure 5A, C). To further analyze tumor composition, immunofluorescence staining was performed to examine the luminal and basal markers, K8 and K14. Analysis revealed that the tumors grown in the ADAM17^WT^ are primarily K8 positive with single K14 cells interspersed throughout the tumor (Figure 5B). Analysis of tumors from the conditional-ADAM17^null^ mice revealed primarily K8-positive cells with a reduction in the numbers of single K14-positive cells (Figure 5D). Together, these findings demonstrate that leukocytespecific ADAM17 deletion leads to reduced tumor onset that correlates with decreased proliferation and reduced numbers of K14-positive cells within the tumors, suggesting that loss of ADAM17 in leukocytes may affect expansion of the basal cell population.

### Loss of leukocyte-specific ADAM17 leads to decreased Cox-2 expression within mammary tumors

**Figure 6:**
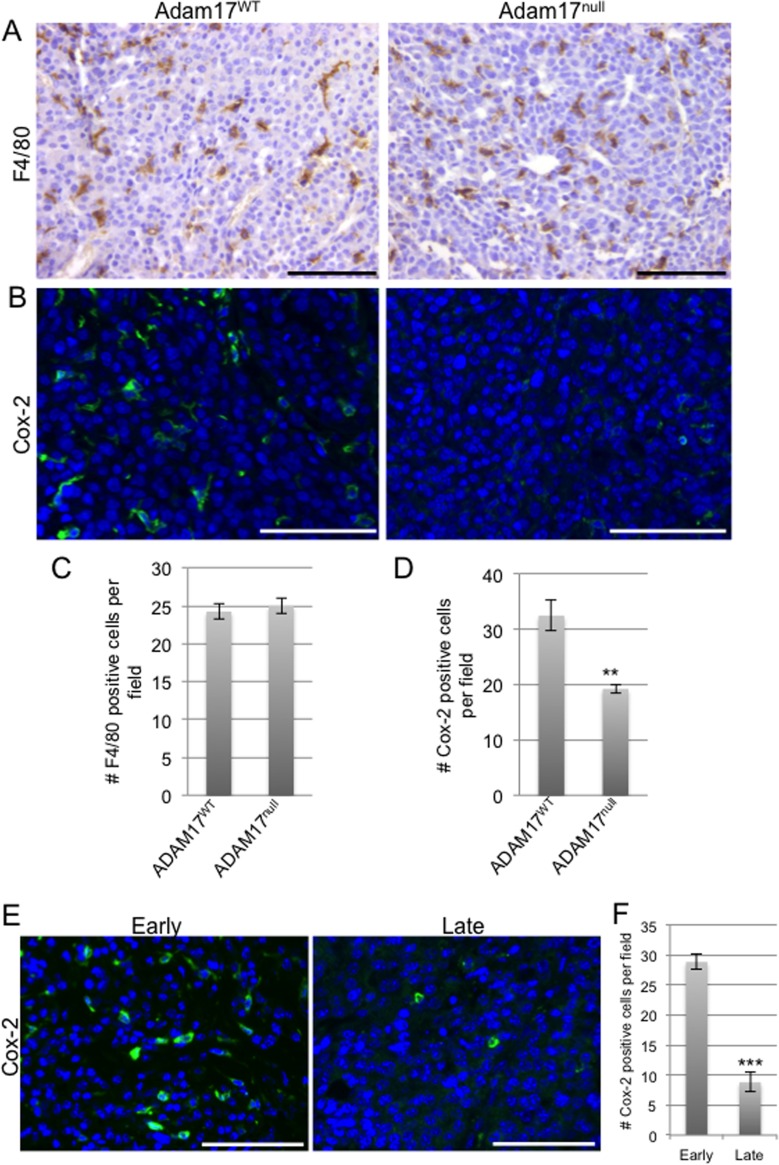
ADAM17 deletion in the hematopoietic compartment leads to reduced Cox-2 expression in developing tumors A) Tumors from Figure 5 were examined for macrophage infiltration by performing immunohistochemical analysis of F4/80 staining (n=3 tumors per genotype). B) Tumors were also examined for Cox-2 expression by immunofluorescence (n=3 tumors per genotype). C,D) Quantification of F4/80 positive (C) and Cox-2 positive (D) cells. E) Staining of Cox-2 in PyMT-derived tumors at either 50 mm3 (early stage) or 1 cm3 (late stage), n=3 tumors per timepoint. F) Quantification of Cox-2 positive cells per field. Scale bars represent 50 μm. ^**^p<0.01, ^***^p<0.001.

Further analysis of the tumors was performed to determine whether loss of ADAM17 in leukocytes affected macrophage infiltration and expression of Cox-2 within the tumors. Analysis of F4/80 staining in the early stage PyMT tumors described above revealed that deletion of leukocyte-specific ADAM17 did not affect macrophage infiltration into the tumor (Figure 6A, 6C). Further studies were performed to assess Cox-2 expression within the tumors. As shown in Figure 6B, numerous Cox-2 positive cells were observed within tumors grown in *ADAM17^WT^* mice. However, there was a significant decrease in the intensity of staining and in the number of Cox-2 positive cells within tumors grown in conditional-*ADAM17^null^* mice (Figure 6B, 6D). Together, these studies demonstrate that ADAM17 is not a key regulator of macrophage recruitment into the tumor microenvironment, but that ADAM17 in leukocytes contributes to Cox-2 expression within mammary tumors.

Because loss of ADAM17 in leukocytes affects tumor onset but not tumor growth rates (Figure 4 and data not shown), Cox-2 expression was assessed in both early and late stage PyMT-derived tumors. Cox-2 expression was found throughout the early stage tumors (Figure 6E). However, reduced numbers of Cox-2 positive cells were found in late stage tumors (Figure 6E-F). These results are consistent with the observation that loss of the ADAM17/Cox-2 axis affects early stage tumorigenesis but not late stage tumor growth.

### Expression of ADAM17 and Cox-2 are linked to reduced relapse free survival in breast cancer patients

**Figure 7:**
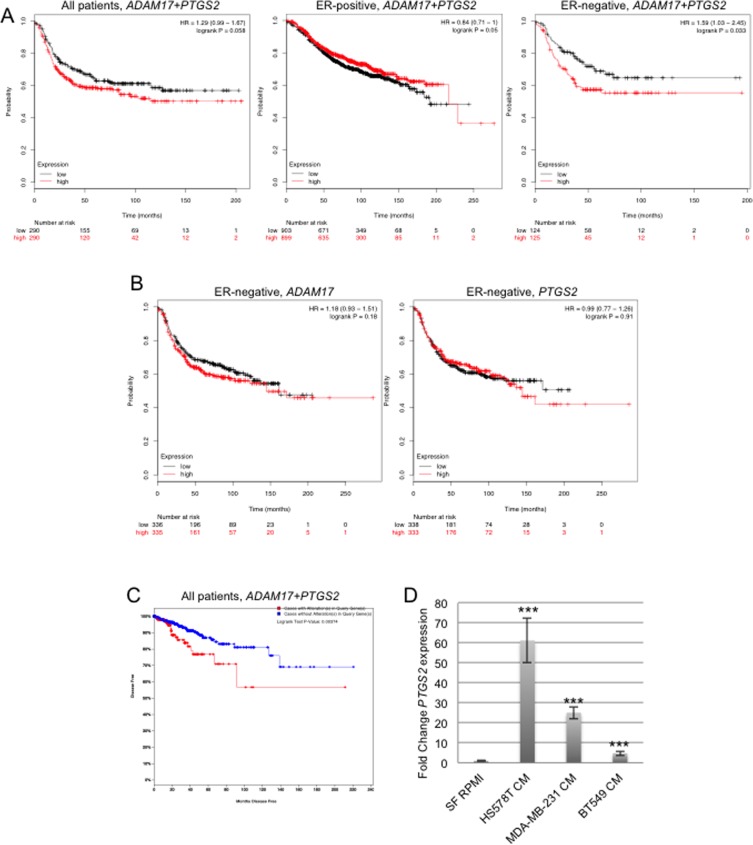
Increased expression of ADAM17 and PTGS2 correlates with reduced disease and relapse free survival A) Analysis of gene expression levels of *ADAM17* and *PTGS2* (Cox-2) using the kmplotter gene expression database demonstrating significantly reduced RFS in estrogen negative breast cancer patients. B) Analysis of *ADAM17* and *PTGS2* expression separately does not demonstrate significantly altered RFS in ER-negative patients. C) Analysis of gene expression levels of *ADAM17* and *PTGS2* the TCGA gene expression database. Graph of disease free survival of patients with alterations in these genes. D) Conditioned medium was collected from the Hs578T, MDA-MB-231 and BT549 cells. PMA-differentiated THP-1 cells were exposed to the conditioned medium, or serum free medium as control, for 18 hours. Expression of *PTGS2* was examined by qRT-PCR. Expression levels were normalized to *CYPB*. ^**^*p<0.001.

To determine the importance of these inflammatory factors in human breast cancer samples, analysis of publically available databases was performed. Gene expression in these databases is based on sequencing of whole tumors, and expression levels of genes specifically in leukocytes are not available thus gene expression analysis can not be performed specifically on tumor associated leukocytes using these databases. However, we rationalized that analysis of these genes in tumors would provide an overview of the potential importance of these factors in the context of breast cancer, regardless of the cell type in which they are expressed. Analysis of the kmplotter database demonstrated that increased mean expression of ADAM17 and PTGS2 was associated with a non-significant trend towards reduced relapse free survival (RFS) in all breast cancer patients (Figure 7A). Further analysis of subsets of breast cancer patients revealed that while there was not a significant reduction in RFS in ER-positive breast cancer patients, a significant reduction in RFS was found for ER-negative breast cancer patients (Figure 7A). Single gene analysis demonstrated no significant differences in RFS based on expression of ADAM17 or PTGS2 alone, suggesting that this effect is specific for patients with increased expression levels of both genes (Figure 7B). Analysis of the TCGA database demonstrated that alterations in *ADAM17* and *PTGS2* were also associated with reduced disease free status (Figure 7C).

Based on these results, further studies were performed to examine the ability of triple negative human breast cancer cells to induce expression of Cox-2 in THP-1-derived macrophages. PMA-differentiated THP-1 cells were exposed to conditioned media obtained from Hs578T, MDA-MB-231 and BT549 cells, which are human triple negative breast cancer cell lines. Soluble factors from all of the cell lines significantly induced expression of Cox-2 in THP-1 cells, although to varying degrees (Figure 7D).

## DISCUSSION

ADAM17 contributes to breast cancer growth and progression through the cleavage and shedding of key soluble factors, including members of the EGF ligand family [17, 18, 31]. However, previous work have focused primarily on ADAM17 in the cancer cells and expression of ADAM17 in cells located within the tumor stroma has not been previously examined. Our findings demonstrate that high levels of ADAM17 expression are found on leukocytes located within the tumor microenvironment. ADAM17 has been linked to the regulation of leukocyte function, including neutrophil recruitment and macrophage efferocytosis [21, 25, 26, 32]. The results presented here also demonstrate that ADAM17 regulates the expression of key inflammatory mediators, including pro- inflammatory cytokines and Cox-2, in tumor associated macrophages. There are a number of ADAM17 substrates that could potentially regulate Cox-2 expression. For example, TNFα, a well-established ADAM17 substrate [19, 35], has been linked to regulation of Cox-2 expression in numerous cell types including fibroblasts, epithelial cells and macrophages [24, 36–39]. ADAM17 substrates include additional factors known to induce inflammatory pathways, including TRANCE/RANKL and CX3CL1 [14] and further studies are required to fully define the ADAM17 substrate that contributes to regulation of Cox-2 expression in macrophages. ADAM17 also regulates shedding of EGF ligands, which have been linked to regulation of Cox-2 expression [40, 41]. Macrophages lack expression of EGFR ([42] and data not shown). Thus, while this may not be the primary mechanism through which ADAM17 regulates Cox-2 in macrophages, it is feasible that shedding of EGF ligands into the tumor microenvironment may regulate Cox-2 expression in surrounding cell types *in vivo*. In conclusion, there are a number of potential mechanisms through which ADAM17 contributes to Cox-2 expression during tumorigenesis.

Cox-2 has previously been linked to tumor associated macrophage function. For example, treatment of tumor associated macrophages with the Cox-2 inhibitor celecoxib reduced M2 polarization and enhanced production of IFNγ, contributing to an antitumor environment [23]. This is further supported by the finding that Cox-2 deletion in myeloid cells using the LysM-Cre model leads to reduced mammary tumor formation in the MMTV-Neu mammary tumor model [27]. Cox-2 expression in tumor associated macrophages has been linked to poor outcome for breast cancer patients and overexpression of Cox-2 in macrophages leads to enhanced growth of mammary tumors in mice [22]. Thus, evidence from both mouse models and human breast cancers support the function of Cox-2 as a key regulator of pro-tumorigenic function in macrophages. As described here, our findings define ADAM17 as an important regulator of Cox-2 expression in macrophages *in vitro* and in mammary tumors *in vivo*.

Based on our findings, we propose that ADAM17- regulated inflammatory mediators, such as Cox-2, are important promoters of tumor formation. Cox-2 inhibition has been considered in the context of prevention. For example, in a recent study, Cox-2 was identified as an independent prognostic factor for ductal carcinoma *in situ* (DCIS) relapse suggesting that it may serve as a targetable biomarker for patients with high risk of DCIS recurrence [43]. Cox-2 has also been linked to progression of DCIS during post-partum involution [44] and more recently to promoting lymphangiogenesis and nodal metastasis in this environment [45]. Several population-based studies have been performed to determine whether potential benefits exist for Cox-2 inhibition as a prevention strategy in cancer patients. While there is a clear association between use of non-steroidal anti-inflammatory drugs (NSAIDs) in some cancers, such as aspirin in colorectal cancer [46], the association between Cox-2 inhibition and breast cancer risk has been less clear. While some epidemiological studies have suggested no benefit for NSAID use in terms of reduction of breast cancer risk overall [47, 48], others have found some reduction in breast cancer risk following long-term aspirin usage [49]. Further subset analysis has suggested that NSAIDs can reduce breast cancer risk in specific patient populations. These include patients with *in situ*, hormone receptor positive and lymph node positive cancers as well as post-menopausal women [50–52]. Thus, further cohort analysis may identify at-risk patients who might benefit from anti-inflammatory prevention strategies. The results described here, as well as our previously published studies focusing on mechanisms of early stage tumor formation, suggest the importance of the Cox-2 pathway during early stages of tumor formation. Using an inducible model of FGFR1 activation, we have previously shown that treatment of mice with celecoxib led to a reduction in the formation of early stage hyperplasias [28, 53]. Therefore, it is feasible that this pathway is important during early stages of tumor formation and that targeting this pathway in early stage lesions may be more effective than during later stages of invasive cancer, at which time the tumors have likely developed multiple mechanisms of driving tumor progression. The identification of high-risk patients with early stage breast lesions that will go on to develop invasive breast cancer has remained a significant challenge within the field. Future studies addressing the presence of macrophage-specific ADAM17 and Cox-2 in early stage lesions, and determining whether these factors correlate with enhanced risk of developing invasive breast cancer could ultimately lead to the development of novel preventive approaches that target these pathways.

To determine whether these pathways are linked to patient outcome, we analyzed gene expression using publically available datasets that were generated from whole tumors. While this precludes our ability to specifically examine gene expression in leukocytes, this type of analysis can help to identify key pathways involved in driving breast cancer progression regardless of the specific cell type involved, which likely provides more utility in a diagnostic setting than analyzing gene expression changes in single cell types. Interestingly, we found that tumors expressing high levels of the factors studied here, including *ADAM17* and *PTGS2* (Cox-2), are associated with reduced relapse free survival, consistent with the prediction that tumors with high levels of inflammatory mediators are more prone to therapeutic resistance and recurrence [54]. Therefore, further studies are warranted to determine whether ER-negative patients will benefit from post-treatment exposure to Cox-2 inhibitors to reduce recurrence.

In summary, our findings implicate ADAM17 as an important regulator of leukocyte function during mammary tumor formation. ADAM17 contributes to the regulation of key inflammatory factors, such as Cox-2, implicating ADAM17 in regulating key protumorigenic pathways. Increased expression levels of these factors within tumors correlates with reduced disease and relapse-free survival, which has implications for treatment of patients with inhibitors of these mediators to prevent recurrence. Inhibitors of Cox-2 are currently used in patients. In addition, the recent development of ADAM17-selective inhibitors, which are well-tolerated in the clinical setting [55, 56], suggests potential use of these inhibitors in the context of breast cancer. The findings from these studies highlight the importance of defining the specific mechanisms through which cells within the tumor microenvironment contribute to tumor growth and progression.

## MATERIALS AND METHODS

### Mice

ADAM17^flox/flox^ (Adam17^tm1.2Bbl^/J) mice and *Vav1-cre* mice (B6.Cg-Tg(Vav1-cre)A2Kio/J) are from The Jackson Laboratory (Bar Harbor, ME). The mice were backcrossed for a minimum of 10 generations to the FVB/N background. The ADAM17^flox/flox^ and *Vav1-cre* mice were then crossed to generate mice lacking leukocyte- specific ADAM17. The ADAM17^fl/fl^/Vav1-cre mice are designated conditional-ADAM17^null^ and the ADAM17^fl/ fl^ mice lacking the Cre transgene, used as controls, are designated ADAM17^WT^. All animal care and procedures were approved by the Institutional Animal Care and Use Committee of the University of Minnesota and were in accordance with the procedures detailed in the Guide for Care and Use of Laboratory Animals.

### Mouse treatments

For tumor induction, 500,000 primary tumor cells derived form MMTV-PyMT mice were injected into fat pads of recipient FVB/N mice (Envigo Laboratories). Mice were monitored for tumor onset by palpation and mice were considered tumor-bearing once tumors reached approximately 100 mm^3^. Tumor growth was measured using calipers and tumor volume was calculated using the following equation: V=(L×W^2^)/2.

### Cell culture

RAW 264.7 and THP-1 cells were obtained and maintained as suggested by American Type Culture Collection (ATCC) (Manassas, VA). THP-1 differentiation was performed using 5 ng/ml phorbol myristate acetate (PMA) (Sigma-Aldrich, St. Louis, MO) for 24 hours. PyMT cells, isolated from tumors generated in MMTV- PyMT mice, were provided by Dr. Felicite Noubissi and Dr. Brenda Ogle and grown in DMEM/F12, 5 μg/ ml insulin (Akron Biotech) and 1 μg/ml hydrocortisone (Sigma-Aldrich), 5 μg/ml EGF (Life Technologies), 5% fetal bovine serum and 1% penicillin-streptomycin (Life Technologies). Bone marrow derived macrophages (BMDMs) were obtained as described previously [57]. All cells were grown at 37°C and 5% CO. BT549, MDA- MB-231 and Hs578T cells were obtained from ATCC and maintained as recommended.

### Cell stimulation

For the human cell lines, subconfluent monolayers were incubated with serum free media for 24 hours to collect soluble factors. Conditioned media samples were collected, filtered and incubated with differentiated THP-1 cells for 18 hours prior to RNA collection. For M1 and M2 stimulation, BMDMs or RAW 264.7 cells were treated with 20 ng/ml LPS (Sigma), IFNγ (R&D Systems), IL-4 (R&D Systems) or IL-13 (R&D Systems) for 18 hours prior to RNA analysis.

### Immunoblot analysis

Cells and tissues were lysed in RIPA buffer as previously described [58]. Immunoblot analysis was performed by incubating membranes overnight at 4°C with the following antibodies: Adam17 (#ab2051, Abcam), GAPDH (#2118, Cell Signaling Technology), Cox-2 (sc-1747, Santa Cruz Biotechnology) and β-tubulin (#2146, Cell Signaling Technology).

### Quantitative reverse transcription-PCR

RNA was extracted from cells using TriPure (Roche) and cDNA was prepared using the qScript cDNA synthesis kit (Quanta Biosciences) according to the manufacturers’ protocols. Quantitative RT-PCR (qRT-PCR) was performed using PerfeCTa SYBR Green (Quanta Biosciences) and the Bio-Rad iQ5 system. The 2^−ΔΔCt^ method [59] was used to determine relative quantification of gene expression and normalized to *cyclophilin B (CYBP)*. Primer sequences are provided in Supplementary Table 1.

### Tissue analysis

Mammary tumors were frozen in OCT or fixed for 2 hours in 4% paraformaldehyde, sectioned and stained with hematoxylin and eosin as described previously [28]. For frozen sections, 5μm thick sections were fixed in acetone for 5′ at room temperature and stained with the following antibodies: K8 (1:200, Developmental Studies Hybridoma Bank, TROMA-1), CD45 (1:100, BD Biosciences, 550539), ADAM17 (1:200, Abcam, ab2051), F4/80 (1:100, BioRad, MCA49RT) and Cox-2 (1:200, Santa Cruz Biotechnology, sc-1747). For staining of paraffin-embedded tissues, the following antibodies and conditions were used. F4/80 (1:100; no antigen retrieval, MCA49RT, BioRad) and BrdU (1:300, sodium citrate antigen retrieval, Abcam, ab6326). For quantification of staining, total numbers of positive cells were counted in at least five 40x images taken from a minimum of three tumors per genotype.

### Analysis of gene expression in human breast cancer databases

Analysis was performed using two publically available repositories, including the cBio portal (http://www.cbioportal.org) for analysis of TCGA datasets and kmplotter (http://kmplot.com/analysis). For the TCGA data, genes were analyzed using the TCGA, Cell 2015 (Breast Invasive Carcinoma) dataset. For kmplotter analysis, RFS was examined in either ER^−^ or ER^+^ cohorts using the mean expression levels of *PTGS2* and *ADAM17*.

### Statistical analysis

Experiments were performed at least three separate times. Statistical analysis was performed using an unpaired student's t-test to compare means. Statistical analysis for percent tumor free data was performed using the log-rank (Mantel-Cox) test. In all figures error bars represent the standard error of the mean.

## SUPPLEMENTARY TABLE


