# Supplementary material for: ADAM17 in tumor associated leukocytes regulates inflammatory mediators and promotes mammary tumor formation
Source: Genes Cancer. 2016 Jul;7(7-8):240–53. doi: 10.18632/genesandcancer.115 (PMC5059114; doi:10.18632/genesandcancer.115)
Supplement: Supplementary file 1 [file ganc-07-240-s001.pdf]

# ADAM17 in tumor associated leukocytes regulates inflammatory mediators and promotes mammary tumor formation – Bohrer et al

**Supplementary Table 1: Primer sequences**

| Gene Name | Sequence |
|---|---|
| Mouse *iNOS* Forward | 5’-GTTCTCAGCCCAACAATACAAGA-3’ |
| Mouse *iNOS* Reverse | 5’-GTGGACGGGTCGATGTCAC-3’ |
| Mouse *ArgI* Forward | 5'-CTCCAAGCCAAAGTCCTTAGAG-3' |
| Mouse *ArgI* Reverse | 5'-AGGAGCTGTCATTAGGGACATC-3' |
| Mouse *Ptgs2* Forward | 5'-TGAGCAACTATTCCAAACCAGC-3' |
| Mouse *Ptgs2* Reverse | 5'-GCACGTAGTCTTCGATCACTATC-3' |
| Mouse *Cyclophilin B* Forward | 5’-TGCAGGCAAAGACACCAATG-3’ |
| Mouse *Cyclophilin B* Reverse | 5’-GTGCTCTCCACCTTCCGT-3’ |
| Mouse *IL-12* Forward | 5'-TGGTTTGCCATCGTTTTGCTG-3' |
| Mouse *IL-12* Reverse | 5'-ACAGGTGAGGTTCACTGTTTCT-3' |
| Mouse *IL-1β* Forward | 5’-GCAACTGTTCCTGAACTCAACT-3’ |
| Mouse *IL-1β* Reverse | 5'-ATCTTTTGGGGTCCGTCAACT-3' |
| Mouse *IL-6* Forward | 5'-TAGTCCTTCCTACCCCAATTTCC-3' |
| Mouse *IL-6* Reverse | 5'-TTGGTCCTTAGCCACTCCTTC-3' |
| Mouse *TNFα* Forward | 5'-CCTCTTCTCATTCCTGCTTGTG-3' |
| Mouse *TNFα* Forward | 5'-TGGGCCATAGAACTGATGAGAG-3' |
| Human *PTGS2* Forward | 5'-GCCTGGGGTGATGAGCAGTT-3' |
| Human *PTGS2* Reverse | 5'-CAGAAGGGCAGGATACAGC-3' |
| Human *Cyclophilin B* Forward | 5'-GAAAGAGCATCTACGGTGAGC-3' |
| Human *Cyclophilin B* Reverse | 5'-GTCTTGACTGTCGTGATGAAGAA-3' |
